# Comprehensive Anesthetic Management for Posterior Mediastinal Tumor Resection in the Prone Position: A Case Report

**DOI:** 10.7759/cureus.85210

**Published:** 2025-06-01

**Authors:** Yui Okune, Hiroki Tateiwa, Tomoko Tsuruno, Yoshifumi Katsumata, Takashi Kawano

**Affiliations:** 1 Department of Anesthesiology and Intensive Care Medicine, Kochi Medical School, Kochi, JPN

**Keywords:** airway compression, artificial pneumothorax, multidisciplinary communication, perioperative planning, posterior mediastinal tumor, preoperative evaluation, prone position

## Abstract

Posterior mediastinal tumors account for a small proportion of mediastinal tumors and are generally less likely to cause perioperative complications than anterior mediastinal tumors. However, large posterior mediastinal tumors or those adjacent to critical thoracic structures can complicate anesthesia management, especially when prone positioning and artificial pneumothorax (AP) are required during video-assisted thoracoscopic surgery. This case report details the successful perioperative management of a 64-year-old woman with a large posterior mediastinal tumor compressing the trachea and left main bronchus, undergoing tumor resection in the prone position. A multidisciplinary team devised a comprehensive plan addressing airway management, cardiovascular stability, and emergency preparedness. Advanced monitoring techniques, including regional oxygen saturation and transesophageal echocardiography, were employed to assess cerebral and cardiovascular stability. Challenges such as airway compression, ventilatory difficulties during AP, and double-lumen tube malposition were effectively managed, ensuring safe surgery and recovery. This case underscores the need for a proactive and multidisciplinary anesthetic strategy employed for a high-risk posterior mediastinal tumor resection in the prone position with bilateral AP. Pre-emptive insertion of sheaths for veno-arterial extracorporeal membrane oxygenation, real-time cerebral and hemodynamic monitoring, and rapid intraoperative response to airway compromise represents a level of detailed planning and adaptability that may offer practical insights for similar cases.

## Introduction

Posterior mediastinal tumors account for a small proportion of mediastinal tumors and are generally less likely to cause perioperative complications than anterior mediastinal tumors due to their anatomical factors [1]. For these reasons, reports on their anesthesia management are rare [2]. However, large posterior mediastinal tumors or those adjacent to critical thoracic structures can cause airway compression and circulatory issues, particularly during anesthesia and surgery [3-6]. Moreover, surgeries involving large tumors often require one-lung ventilation (OLV), which can further challenge oxygenation and ventilation. Furthermore, the potential for complications is heightened by the repositioning. A lateral or supine position is commonly chosen for posterior mediastinal tumor surgery [3]. However, the prone position may be necessary for thoracoscopic surgery of posterior mediastinal tumors to improve the operative field [7]. In addition, artificial pneumothorax (AP) is often provided for the same purpose. However, these conditions can exacerbate airway compression and circulatory instability.

While thoracoscopic surgery is less invasive and advantageous for postoperative recovery, these unique requirements of posterior mediastinal tumor surgery present challenges for anesthesiologists. Specific anesthetic concerns in such cases include: (1) airway compression from the tumor, leading to difficulties in intubation and ventilation; (2) ventilatory compromise during OLV and AP, particularly in the prone position; (3) hemodynamic instability due to tumor proximity to major vessels and body positioning; and (4) risks of cerebrovascular complications, especially when the tumor abuts the aortic arch or subclavian artery.

Given these complexities, a multidisciplinary approach is crucial. Collaboration among anesthesiologists, thoracic surgeons, and other specialists helps in devising comprehensive perioperative plans that address potential complications and ensure patient safety. This case report underscores the importance of such an approach, detailing the successful management of a patient undergoing thoracoscopic surgery for a large posterior mediastinal tumor in the prone position. It underscores the need for thorough preoperative evaluation, meticulous planning, and multidisciplinary communication to minimize risks and optimize outcomes.

## Case presentation

**Figure 1:**
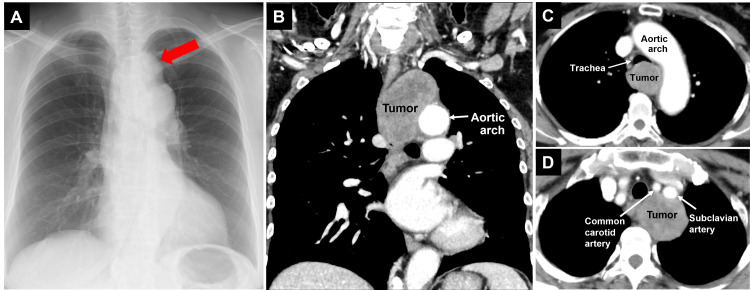
Chest radiograph and computed tomography image before surgery Chest radiograph showing a mass shadow above the first left arch (red arrow in A). A 4.6×4.3×6.5 cm posterior mediastinal tumor was compressing the trachea and left main bronchus, causing approximately 50% stenosis at the tracheal carina (B, C). The tumor was in contact with the esophagus, thoracic vertebrae, aortic arch, left subclavian artery, and azygos venous arch, though the invasion into them was not obvious (B-D).

**Figure 2:**
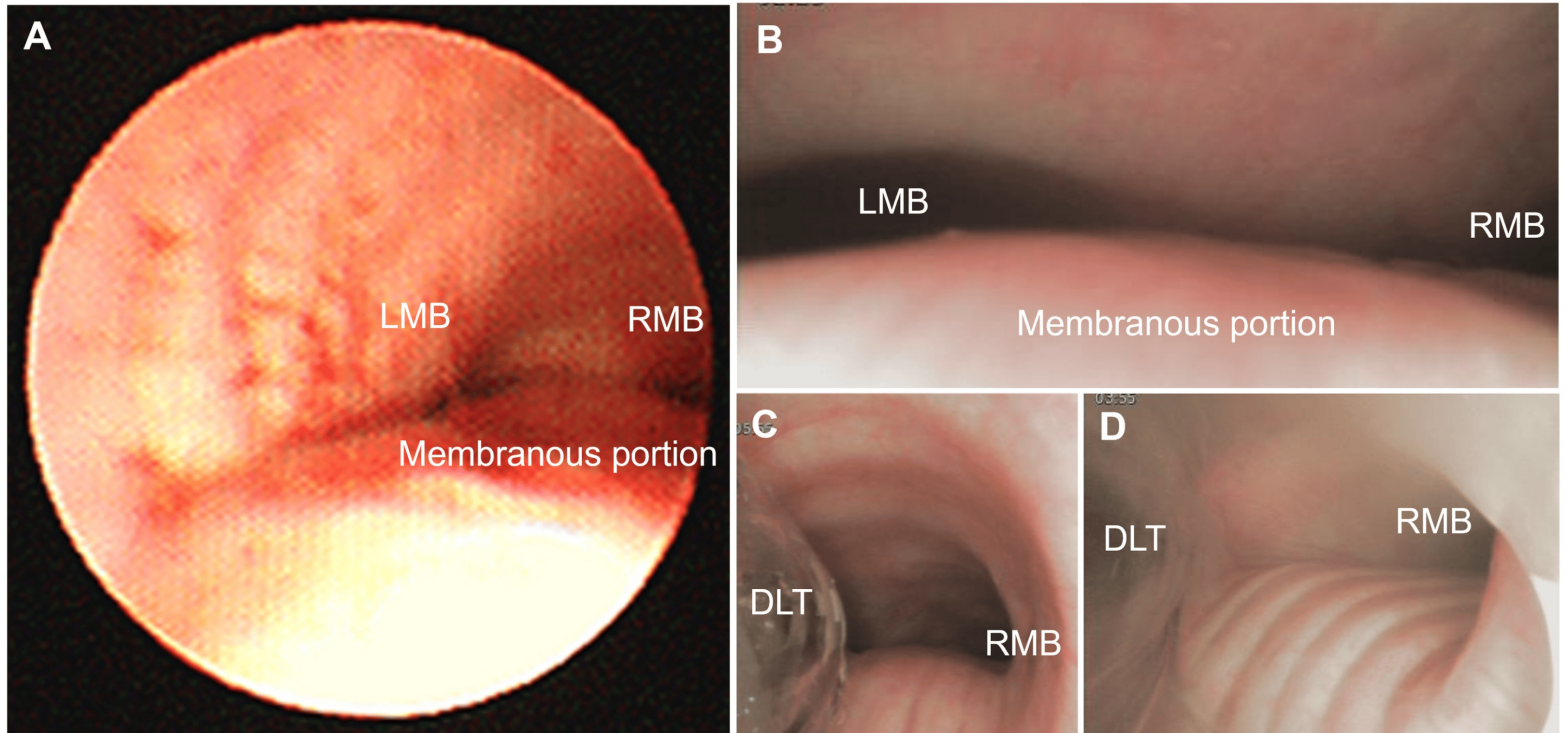
Bronchoscopy before and during surgery (A) The membranous portion of the trachea was compressed by the tumor, causing the stenosis of LMB before surgery. (B) The stenosis of LMB was detected before surgery. (C) The DLT was inserted into the LMB. (D) No airway obstruction before extubation. DLT, double-lumen tube; LMB, left main bronchus; RMB, right main bronchus

A 64-year-old woman was found to have a mass shadow above the first left arch on a chest radiograph taken at a medical checkup (Figure 1A). The patient had no symptoms. Contrast-enhanced computed tomography revealed a 4.6×4.3×6.5 cm posterior mediastinal tumor compressing the trachea and left main bronchus (LMB), causing approximately 50% stenosis at the tracheal carina (Figures 1B, 1C). In addition, the tumor was in contact with the esophagus, thoracic vertebrae, aortic arch, left subclavian artery, and azygos venous arch, though the invasion into them was not obvious (Figures 1B-1D). Bronchoscopy confirmed LMB compression on the membranous portion of the trachea (Figure 2A). Preoperative blood tests, pulmonary function tests, and echocardiography were unremarkable. Magnetic resonance angiography showed no cerebrovascular abnormalities.

Preoperative planning

A multidisciplinary conference involving anesthesiology, thoracic and cardiovascular surgery, gastroenterology, nursing, and clinical engineering developed a perioperative plan addressing airway management, circulatory monitoring, and emergency preparedness. We decided to monitor regional oxygen saturation (rSO_2_) to assess cerebral blood flow and transesophageal echocardiography (TEE) to evaluate tumor invasion into major vessels. Preoperative imaging, bronchoscopy, and rSO_2_ confirmed tracheal and LMB compression. However, since the tumor was located in the posterior mediastinum, the risk of worsened airway obstruction following induction of anesthesia was considered low. Nevertheless, in preparation for ventilatory failure, sheaths for veno-arterial extracorporeal membrane oxygenation (VA-ECMO) were planned to be inserted into the femoral artery and vein after induction of anesthesia. Additionally, anesthetic agents were carefully chosen to allow for pharmacological reversal if necessary. If intubation could not be performed due to tumor-related airway narrowing, fiberoptic-guided intubation in the supine or lateral position was planned. Based on imaging findings and the fact that the posterior mediastinal tumor was located dorsal to the trachea, it was anticipated that even if airway compression increased after induction, advancement of the tube past the stenosis would still be possible. Therefore, a relatively rigid double-lumen tube (DLT) was chosen to facilitate secure advancement through the compressed segment. VA-ECMO initiation was planned if oxygenation or ventilation could not be maintained.

Induction of anesthesia

**Figure 3:**
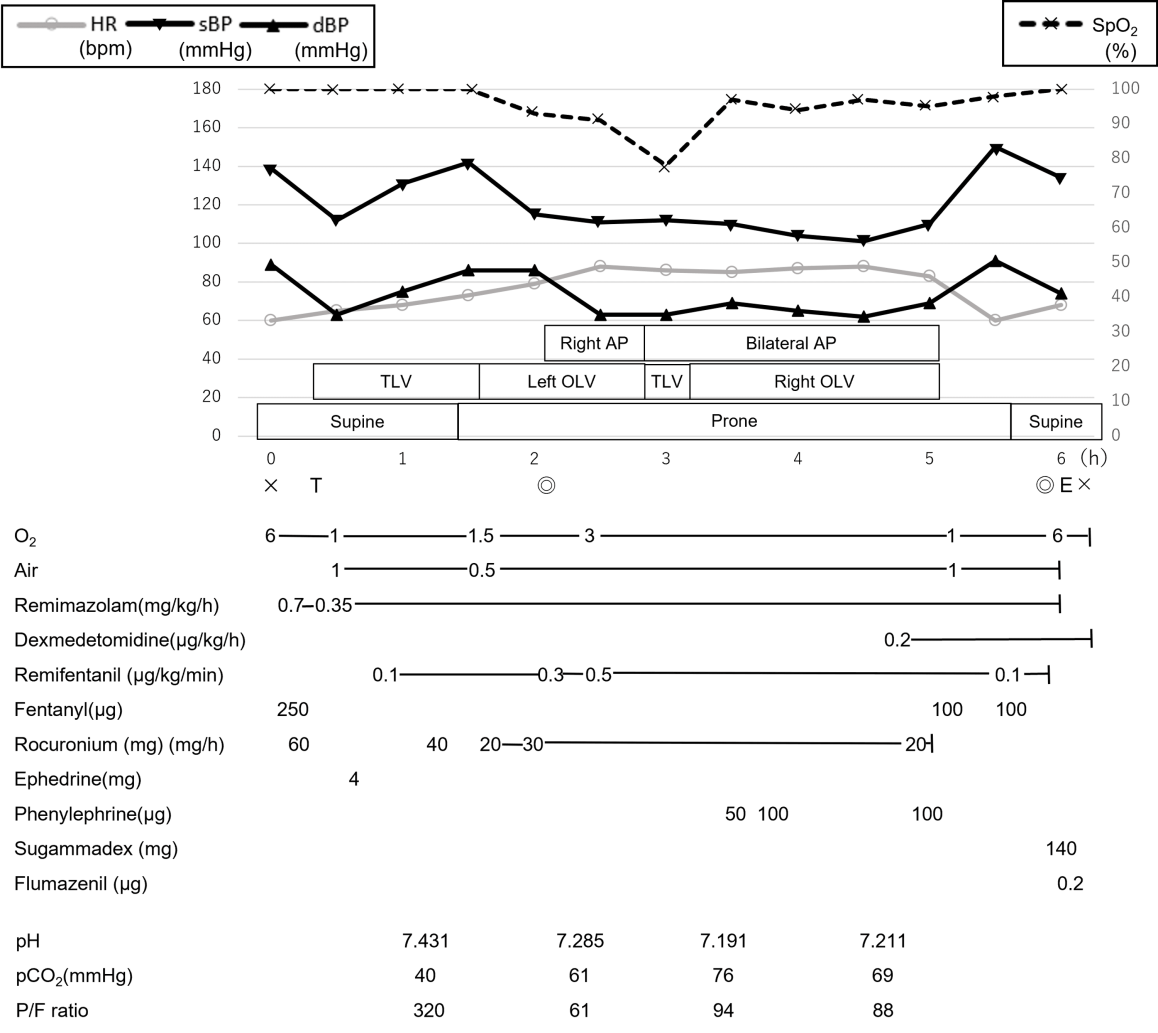
The anesthesia record Cross marks, the beginning and end of anesthesia; T, intubation; E, extubation; double circles, the beginning and end of surgery AP, artificial pneumothorax; dBP, diastolic blood pressure; HR, heart rate (scale on the left side); OLV, one-lung ventilation; sBP, systolic blood pressure; SpO₂, saturation of percutaneous oxygen (scale on the right side); TLV, two-lung ventilation

**Figure 4:**
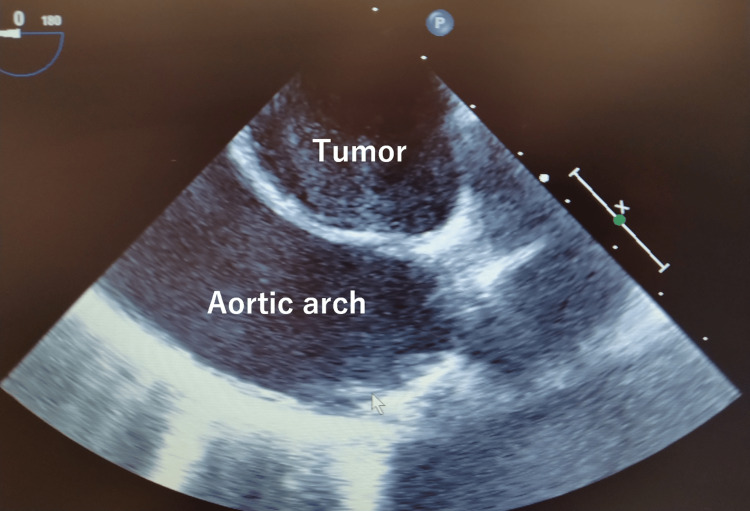
TEE showing that the tumor was mobile and did not invade the aortic arch (after induction of anesthesia) TEE, transesophageal echocardiography

The anesthesia record is shown in Figure 3. The patient's general condition was stable in the supine position. In addition to the standard monitor, rSO_2_ was measured with O3^®^ Regional Oximetry (Masimo Corporation, Irvine, CA, USA). After securing venous access and arterial line, anesthesia was induced with a continuous infusion of remimazolam and a single injection of fentanyl. After the loss of consciousness, the remimazolam dose was reduced, and rocuronium was administered. Mask ventilation was performed easily. Endotracheal intubation was performed using a 32 Fr Coopdech DLT^®^ (Daiken Medical Company Limited, OSA, JPN). Bronchoscopy revealed compression of the membranous portion of the trachea and the stenosis of LMB due to the tumor as detected before surgery (Figure 2B). Using the bronchoscope inserted into the LMB as a guide, the DLT was gently advanced and inserted without resistance (Figure 2C). No hypoxemia occurred during the induction of anesthesia. A central venous route was inserted into the right internal jugular vein, and an arterial line was inserted into the other radial artery to enable observation of the difference in blood pressure between the left and right sides. The TEE was placed as planned, and it was confirmed that the tumor was mobile and did not invade the aortic arch and left subclavian artery (Figure 4). Sheaths into the femoral artery and vein for VA-ECMO were inserted by a cardiovascular surgeon. When repositioning to the prone position, there was no significant decrease in ventilation volume, rSO_2_ values, blood flow in the left subclavian artery on Doppler ultrasound, and no difference in blood pressure between the left and right sides.

Intraoperative management and postoperative course

Anesthesia was maintained with a continuous infusion of remimazolam and remifentanil. Initially, gastrointestinal surgeons performed right thoracoscopic surgery with right AP under left OLV. At this stage, an arterial blood gas analysis revealed the following: pH: 7.285, partial pressure of carbon dioxide (PaCO_2_): 61 mmHg, and PaO_2_/FiO_2_ (P/F) ratio: 61. Subsequently, thoracic surgeons initiated left thoracoscopic surgery with left AP in addition to the pre-existing right AP under two-lung ventilation (TLV). At that time, a sudden decrease in saturation of percutaneous oxygen (SpO_2_) and ventilation volume was observed. We promptly informed the surgeons about it, and the operation and the bilateral AP were paused. Bronchoscopy revealed that the tip of the DLT had migrated out of the LMB into the trachea. The DLT position was immediately corrected, resulting in an improvement in both SpO_2_ and ventilation volume. Consequently, the right OLV with bilateral AP was reinitiated. At this point, an arterial blood gas analysis revealed the following: pH: 7.191, PaCO_2_: 76 mmHg, and P/F ratio: 94. Although PaCO_2_ was high and pH was less than 7.2, surgery was continued by adjusting the ventilation settings. The tumor resection was successfully completed without any complications. At this point, an arterial blood gas analysis revealed the following: pH: 7.211, PaCO_2_: 69 mmHg, and P/F ratio: 88. No issues were encountered during the patient’s repositioning to the supine position. Additionally, the final bronchoscopy confirmed no airway obstruction (Figure 2D). Sugammadex was administered based on the train-of-four (TOF) monitor results, and the TOF ratio recovered to 100%. Remimazolam and remifentanil infusion were discontinued, and flumazenil was administered. Spontaneous eye-opening and breathing were confirmed, followed by the removal of the DLT in the operating room. The patient’s postoperative course in the intensive care unit was uneventful, and the patient was discharged to the general ward the next day in a stable condition.

## Discussion

Surgery for anterior mediastinal tumors has been reported to cause life-threatening airway collapse even under spontaneous ventilation during general anesthesia (GA) [8]. In contrast, surgery for posterior mediastinal tumors poses a lower risk of airway compromise in the supine position since the tumor does not directly overlap the airway [1,3]. On the other hand, caution is required when repositioning to the prone position. A lateral or supine position is commonly chosen for posterior mediastinal tumor surgery [3]. However, the prone position may be necessary for thoracoscopic surgery of posterior mediastinal tumors to improve the operative field, as the gravitational shift of the lungs into the mediastinum or dorsally in the supine or lateral position can obscure the surgical field [7,9].

For surgery with AP in the prone position, TLV using a single-lumen tube provides several advantages over OLV using a DLT. For instance, the previous method improves safety and ease of induction and maintenance of anesthesia and provides better oxygenation [9]. However, the posterior wall of the trachea is unsupported by cartilage and is vulnerable to external pressure [10]. In this case, the tumor was compressing the posterior wall of the trachea, which could have further narrowed the airway in the prone position. For such cases, DLT is the preferred airway management technique to mitigate the risk of obstruction [11].

During the surgery with bilateral AP under TLV, ventilation volume and oxygenation decreased due to the malposition of the DLT.　Adjusting the position of the DLT promptly restored ventilation volume and oxygenation, allowing the surgery to proceed with bilateral AP under right OLV. Notably, oxygenation was better with bilateral AP under right OLV than with right AP under left OLV. Bilateral AP resulted in better oxygenation than one-sided AP, likely because external pressure on the pulmonary alveoli excluded the non-ventilated lung, improving pulmonary shunt fraction and increasing PaO_2_ [9,12].

The preoperative imaging showed that the tumor was in contact with the aortic arch and the left subclavian artery. Tumor invasion into these arteries could lead to arterial damage, circulatory failure, or necessitate vascular repair, including graft replacement. Real-time TEE during surgery can reveal findings that are not apparent on preoperative imaging, guiding treatment plans, and identifying the causes of circulatory instability [3,4]. In this case, TEE confirmed infiltration of the tumor into the arteries, facilitating safe surgical progression.

In this case, sheaths for VA-ECMO were inserted after the induction of anesthesia in preparation for potential respiratory failure in the prone position or circulatory failure during surgery. Additionally, a bed was reserved nearby during surgery to enable immediate repositioning to the supine position and VA-ECMO initiation if needed. A cardiovascular surgeon and a perfusion team were present throughout the surgery. These arrangements were made to ensure that VA-ECMO could be initiated without delay in the event of critical deterioration. Previous reports have highlighted the necessity of cardiopulmonary bypass during the induction of anesthesia for patients with posterior mediastinal tumors [4]. For high-risk patients, depending on the tumor’s characteristics and location, careful preoperative planning and readiness to use devices are essential. Intraoperatively, some degree of ventilatory deterioration, particularly during AP in the prone position, was anticipated. A pH of 7.20 was set as the threshold for acceptable respiratory acidosis, if oxygenation (SpO_2_ >90%) and rSO_2_ remained stable [13]. In cases where ventilatory adjustments failed to achieve improvement, temporary discontinuation of OLV was planned in consultation with the surgical team. In the present case, ventilation improved with the adjustment of ventilator settings alone. However, if ventilatory failure had persisted despite these measures, conversion to the supine position and initiation of VA-ECMO were preoperatively discussed as part of the contingency strategy.

Given the tumor’s contact with the left subclavian artery, there were concerns about cerebrovascular complications. In the prone position, compression of the tumor may cause stenosis or occlusion of the left subclavian artery and left common carotid artery, which may lead to cerebrovascular complications such as cerebral infarction. Monitoring rSO_2_ is effective for the early detection of decreased cerebral blood flow [14]. However, the subclavian artery is mainly involved in the occipital blood flow via the vertebral artery, and rSO_2_ alone is insufficient for monitoring. Therefore, in this case, Doppler ultrasound was used in addition to rSO_2_, and special attention was given to whether there was a decrease in blood flow of the left subclavian artery during repositioning. During GA, there was no decrease in rSO_2_ values and blood flow in the left subclavian artery, and no difference in blood pressure between the left and right sides of the upper limbs was observed. Cerebrovascular complications were absent throughout the perioperative period. The above problems and management strategies are summarized in Table 1.

**Table 1:** Clinical pearls based on the ABCD framework AP, artificial pneumothorax; CNS, central nervous system; DLT, double-lumen tube; OLV, one-lung ventilation; rSO₂, regional oxygen saturation; TEE, transesophageal echocardiography; VA-ECMO, veno-arterial extracorporeal membrane oxygenation

ABCD	Clinical issue	Management strategy
A: Airway	Tracheal and bronchial compression	Pre-induction imaging and bronchoscopy
		Pre-induction insertion of VA-ECMO sheaths
		Selection of a relatively rigid DLT for secure advancement
		Use of anesthetic agents that allow for pharmacological reversal
B: Breathing	Ventilatory failure	Pre-induction insertion of VA-ECMO sheaths
		Adjustment of ventilation settings
		Predefined plan for temporary cessation of OLV or AP if necessary
	Malposition of DLT	Continuous monitoring with SpO_2_ and bronchoscopy
C: Circulation	Tumor invasion or compression of major thoracic vessels	Pre-induction insertion of VA-ECMO sheaths
		TEE to evaluate vessel invasion and real-time hemodynamic stability
D: CNS dysfunction	Cerebral hypoperfusion due to compression of major aortic branches	Multimodal monitoring using rSO_2_ and Doppler ultrasound of the subclavian artery

Although risk factors for perioperative complications and an algorithm for anesthetic management of mediastinal tumors have been suggested [2], no standardized guidelines are currently available. Furthermore, as surgical techniques advance, such as video-assisted procedures, anesthesia strategies must be adapted accordingly. Therefore, comprehensive preoperative evaluation, meticulous perioperative planning, and close multidisciplinary communication are essential for achieving successful outcomes [2,4,5].

This case provides several instructive points that may not be well documented in previous reports. First, thoracoscopic resection of a large posterior mediastinal tumor in the prone position with bilateral AP is relatively rare and poses unique anesthetic challenges. Second, this report demonstrates the utility of pre-emptive VA-ECMO preparation when airway compromise and ventilatory failure are anticipated even in posterior mediastinal tumors. Third, the use of multimodal monitoring, including rSO_2_, TEE, and Doppler ultrasound, contributed to intraoperative safety in a high-risk patient. These strategies, planned and implemented through multidisciplinary collaboration, may help inform anesthetic approaches in similarly complex cases.

## Conclusions

This case underscores the successful anesthetic management of a patient undergoing posterior mediastinal tumor resection, which is a procedure associated with potential respiratory, hemodynamic, and neurological risks. In addition to standard comprehensive planning, this case underscores the value of specific strategies that are rarely reported in similar contexts: prone thoracoscopic resection with OLV under bilateral AP, proactive preparation for VA-ECMO, and the use of multimodal monitoring, including rSO_2_ and TEE. These approaches may offer practical guidance for anesthesiologists managing complex posterior mediastinal tumors.
